# Endogenous Biomarkers for SLC Transporter-Mediated Drug-Drug Interaction Evaluation

**DOI:** 10.3390/molecules26185500

**Published:** 2021-09-10

**Authors:** Yang Li, Zahra Talebi, Xihui Chen, Alex Sparreboom, Shuiying Hu

**Affiliations:** Division of Pharmaceutics and Pharmacology, College of Pharmacy & Comprehensive Cancer Center, The Ohio State University, Columbus, OH 43210, USA; li.10991@osu.edu (Y.L.); talebi.9@osu.edu (Z.T.); chen.9570@buckeyemail.osu.edu (X.C.); sparreboom.1@osu.edu (A.S.)

**Keywords:** membrane transporters, endogenous biomarkers, drug-drug interactions, drug development

## Abstract

Membrane transporters play an important role in the absorption, distribution, metabolism, and excretion of xenobiotic substrates, as well as endogenous compounds. The evaluation of transporter-mediated drug-drug interactions (DDIs) is an important consideration during the drug development process and can guide the safe use of polypharmacy regimens in clinical practice. In recent years, several endogenous substrates of drug transporters have been identified as potential biomarkers for predicting changes in drug transport function and the potential for DDIs associated with drug candidates in early phases of drug development. These biomarker-driven investigations have been applied in both preclinical and clinical studies and proposed as a predictive strategy that can be supplanted in order to conduct prospective DDIs trials. Here we provide an overview of this rapidly emerging field, with particular emphasis on endogenous biomarkers recently proposed for clinically relevant uptake transporters.

## 1. Introduction

**Figure 1:**
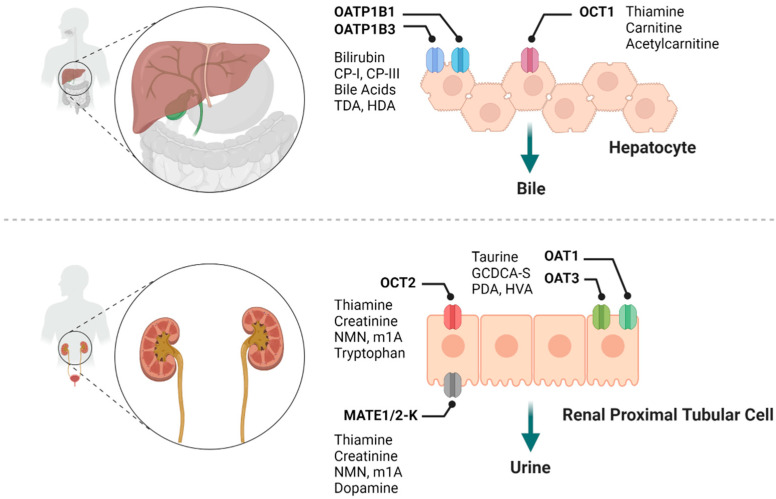
Summary of various drug transporters and their potential endogenous biomarkers. CP-I, coproporphyrin I; CP-III, coproporphyrin III; TDA, tetradecanedioate; HDA, hexadecanedioate; NMN, *N*^1^-Methylnicotinamide; m^1^A, *N*^1^-Methyladenosine; GCDCA-S, glycochenodeoxycholate-3-sulfate; PDA, pyridoxic acid; HVA, homovanillic acid.

Membrane transporters of the ATP-binding cassette (ABC) and solute carrier (SLC) super families are now widely recognized as important determinants of the absorption, distribution, metabolism, and excretion of many xenobiotic compounds, and for many of these transporters, physiological substrates have been identified [1,2]. The increasing application of polypharmacy regimens, in conjunction with current regulatory requirements, demands that new investigational drugs are evaluated as substrates or inhibitors of select transporters in the early stages of development in order to predict the liability for drug-drug interactions (DDIs) [3]. In the last few years, significant progress has been made in evaluating endogenous metabolites as potential clinical biomarkers to predict transporter-mediated DDIs, in particular those in which the drug of interest is a suspected perpetrator [4]. This is based on the tenet that such drugs, once administered, can reach levels sufficiently high to inhibit transporters that fulfill a role in the movement of known endogenous substrates, and that this inhibition is accompanied by measurable, acute, and temporal increases in the systemic concentration of those substrates. Metabolomics, a systems-wide omics analysis of endogenous metabolites in biological samples, is one commonly used approach to identify the endogenous substrates of drug transporters that could serve as a biomarker in humans and other species [5]. In combination with the use of animals with a genetic deficiency of a transporter or human subjects with genetic predisposition of altered transporter function, these metabolomics studies can facilitate the discovery of novel endogenous substrates of drug transporters [6,7]. Following appropriate in vitro and in vivo validation, such substrates can then be explored as a potential predictive biomarker and be further used in vivo to assess the potential for transporter-mediated DDIs. In the current article, we provide an overview of this rapidly emerging field of endogenous transporter biomarkers, focus on its application to predict drug transporter-mediated DDIs in humans associated with established organic anion and cation transporters (Figure 1), and address current challenges and limitations in this field.

## 2. Organic Anion Transporters

Organic anion transporters (OATs), along with organic anion-transporting polypeptides (OATPs), organic cation transporter (OCTs), organic cation and carnitine transporters (OCTNs), and multidrug and toxin extrusions proteins (MATEs) [8], are a SLC family of transporters that have been most commonly associated with the transport of xenobiotic drugs. These transporters are expressed in key organs of elimination such as kidney and liver, as well as in other organs such as the intestine and brain, and play an important role in maintaining normal homeostasis, mediating drug absorption and disposition properties [9]. OATPs and OATs are the major organic anion-type transporters, where OATPs typically transport relatively large and fairly hydrophobic organic anions, while OATs transport smaller and more hydrophilic organic anions [9]. Recent preclinical and clinical studies have identified and validated several endogenous substrates as potential biomarkers for organic anion-type transporters (Table 1).

### 2.1. OATP1B1 and OATP1B3

Organic anion-transporting polypeptides comprise a family of transporters expressed in various tissues [42]. In humans, OATP1B1 [*SLCO1B1*] and OATP1B3 [*SLCO1B3*] (collectively referred to as OATP1B hereafter) are partially redundant transporters that are highly expressed on the sinusoidal membrane of hepatocytes and play key roles in the hepatic uptake of drugs [43,44,45]. Inhibition of OATP1B can lead to defective elimination, result in increases in plasma concentration of drugs that are substrates of these transporters, delayed clearance, and ultimately increase the risk of therapy-related side effects [45,46]. Both the US Food and Drug Administration (FDA) and European Medicines Agency (EMA) guidelines require under certain conditions that in vitro studies are performed to evaluate whether investigational new drugs are potential substrates or inhibitors of OATP1B [47,48]. A multitude of endogenous substrates of OATP1B have been identified in the past few years and some of these have been proposed as potential biomarkers in conjunction with clinical evaluations of OATP1B-mediated DDIs [4,5,49].

#### 2.1.1. Bilirubin

Bilirubin is the end product of heme breakdown and its hepatocellular uptake is at least partially dependent on OATP1B1 and OATP1B3 [50]. After bilirubin enters the liver, it is detoxified by UGT1A1-mediated conjugation to form bilirubin-glucuronide and bilirubin-di-glucuronide. Most of the bilirubin glucuronides are subsequently excreted into bile by MRP2 [*ABCC2*], but some of the conjugated bilirubin is effluxed back into the sinusoidal blood by MRP3 [*ABCC3*] and enter a shuttling-loop (called ‘hepatocyte-hopping’) and be taken up by neighbouring hepatocytes in an OATP1B-dependent manner [50,51]. The role of OATP1B1 and OATP1B3 in the hepatic uptake of conjugated and unconjugated bilirubin has been confirmed by several preclinical and clinical studies. In vitro studies showed that OATP1B1 and OATP1B3 substantially contribute to unconjugated bilirubin uptake in adult hepatocytes [10,11]. In vivo studies using a Slco1b2 knockout (Oatp1b2-null) mouse model further revealed that Oatp1b2, the single murine orthologue of human OATP1B1 and OATP1B3, is important in the hepatic uptake of unconjugated and conjugated bilirubin [52,53]; studies using humanized OATP1B1, OATP1B3 transgenic mice showed partial or complete rescue of increased plasma bilirubin levels [54,55]. Consistent with these preclinical observations, it has been reported that carriers of impaired function variants in the genes encoding OATP1B1 and OATP1B3 have significantly elevated levels of unconjugated bilirubin [56,57]. More interestingly, subjects with rotor syndrome manifest clinically with extensive hyperbilirubinemia, and this phenotype has been causally connected with a rare condition associated with a complete loss of function of both OATP1B1 and OATP1B3 [58,59,60].

The utility of using bilirubin and its conjugated forms as endogenous biomarkers to assess OATP1B-mediated DDIs potential has been extensively evaluated. In rats administered 20 or 80 mg/kg rifampin, a known OATP1B inhibitor, the plasma levels of total bilirubin and conjugated bilirubin were significantly and dose-dependently elevated compared with rats receiving vehicle alone [61]. In cynomolgus monkeys, after single oral administration of 18 mg/kg rifampin, a 2.4-, and 3.0-fold elevation of unconjugated and conjugated bilirubin has been observed [62]. Furthermore, administration of rifampin (300 or 600 mg) in healthy volunteers increased the concentrations of direct bilirubin in a dose-dependent manner; the AUC_0–24h_ of direct bilirubin was 2.3- and 3.5-times higher than the control values [13]. This finding was independently verified in another study involving healthy subjects receiving 150, 300, or 600 mg rifampicin [12]. Although bilirubin has been shown to be sensitive to OATP1B inhibitors and the alteration in bilirubin level correlates well with changes observed in other xenobiotic OATP1B substrates, such as the drugs atorvastatin, pitavastatin, and valsartan [12], it remains unclear to what extent other transporters (ABCC2, ABCC3) and enzymes (UGT1A1) of relevance contribute to the elimination of bilirubin, which therefore diminishes its specificity as a biomarker. It is worth pointing out that serum bilirubin levels are also used as a biomarker for liver injury, and any changes in bilirubin levels must be carefully evaluated, and the data need to be analysed in a manner that can distinguish between reversible inhibition of hepatic OATP1B, ABCC2, ABCC3, and/or UGT1A1 versus liver injury [49].

#### 2.1.2. Coproporphyrin I and III

Coproporphyrins (CPs) are by-products of heme synthesis, and two of these, CP-I and CP-III, are of particular interest in the context of functional OATP1B modulation; CP-I and CP-III are metabolically stable and eliminated in bile and urine, respectively, as intact forms in humans [63]. CP-I and CP-III are relatively selective substrates for OATP1B1 and OATP1B3 [14,64]. In mice study, CPs level in plasma and urine were significantly increased in Oatp1a/1b gene cluster knockout mice (Oatp1a/1b^–/–^), compared with wild-type animals (7.1- to 18.4-fold) [64]. In cynomolgus monkey, administration of OATP1B inhibitors cyclosporin A (100 mg/kg, oral) or rifampicin (15 mg/kg, oral) increased plasma levels of CP-I and CP-III by 2.6- and 5.2-fold, 2.7- and 3.6-fold, respectively [64]. Although CP-I and CP-III are relative specific OATP1B substrates, none of them are transported by OCT1 [*SLC22A1*], OCT2 [*SLC22A2*], OAT3 [*SLC22A8*], or NTCP [*SLC10A1*]; CP-III is identified to be OATP2B1 substrate [14], which leads to the hypothesis that CP-I might reflect OATP1B function more precisely. This concept is further supported by data obtained from healthy volunteers, in which the plasma concentration of CP-I was increased most in subjects with homozygous carriers of the OATP1B1*15 [18,65]. Consistently, CP-I plasma level were not altered by administration of agents not known to inhibit OATP1B, such as itraconazole and diltiazem [66,67].

The utility of CP-I and CP-III as suitable endogenous clinical biomarkers of OATP1B function has been evaluated in several studies. In healthy subjects with a South Asian Indian background, the administration of 600 mg of rifampicin in combination with 5 mg of rosuvastatin, resulted in 4.0- and 3.3-fold increase in plasma AUC levels of CP-I and CP-III, respectively, when compared with rosuvastatin given alone [16], these changes in response to rifampin were consistent with observations obtained from another study with mixed ethnicities [66]. It is important to note that the increase in CP-I in response to rifampin in healthy subjects was greater than several other potential OATP1Bs biomarkers measured simultaneously [12,23,68]. The magnitude effect of CP-I alteration suggests that it is sensitive to differentiate weak, moderate, and strong OATP1B inhibitors reflected by altered plasma level of CP-I. This hypothesis was independently verified by Kunze et al. in which strong (≥5 fold AUC increases; e.g., rifampicin), moderate (2–3 fold AUC increase; e.g., simeprevir), and weak (no change; e.g., the investigational agent JNJ-A) OATP1B inhibitors were used to test CP-I sensitivity [69]. The superiority of CP-I over CP-III as an OATP1B1 endogenous biomarker was recently supported in a clinical study where CP-I C_max_ ratio and AUC ratio relative to baseline were correlated with increasing exposure of glecaprevir (GLE), a drug known to cause clinical inhibition of OATP1B1/1B3, whereas only modest correlation between GLE exposure and CP-III C_max_ ratio but no correlation with CP-III AUC ratio were observed [15]. More recently, CPs have been applied as endogenous OATP1B biomarkers to elucidate in vivo inhibition potency of the anticancer drug, paclitaxel, a known inhibitor of OATP1B1 and OATP1B3, in patients with non–small cell lung cancer [17]. These collective studies have provided compelling evidence that CP-I is an emerging promising endogenous biomarker with higher selectivity and sensitivity to predict the risk of OATP1B transporter-mediated DDIs in humans.

#### 2.1.3. Bile Acids

Bile acids emulsify dietary fats, eliminate cholesterol, and clear hepatic catabolites. Bile acids are synthesized in the liver by cytochrome P450-mediated oxidation of cholesterol and are stored in the gall bladder and released into the intestinal lumen via the bile duct, reabsorbed in the ileum, and returned to the liver, thus completing the enterohepatic circulation [70,71]. The primary bile acids are synthesized and conjugated in the liver, subsequently secreted into the bile. In the intestine, primary bile acids are deconjugated and dehydroxylated by enzymes originating from the gut microflora to form the secondary bile acids. Via enterohepatic circulation, the secondary bile acids are transported to the liver and recycled. Hepatic uptake, the last step in enterohepatic circulation, is a key process in regulating the amount of circulating bile acids. Several primary and secondary bile acids have been identified as OATP1B transporter substrates. As shown from a metabolomics study using Oatp1b2-null mice, levels of unconjugated bile acids were 3- to 45-fold higher than in wild-type mice, including β-muricholic acid (βMCA, 45-fold), cholic acid (CA, 38-fold), α-muricholic acid (αMCA, 25-fold), hyodeoxycholic acid (HDCA, 15-fold), ursodeoxycholic acid (UDCA, 11-fold), chenodeoxycholic acid (CDCA, 3-fold), and deoxycholic acid (DCA, 2.3-fold) [72]. In vitro studies confirmed that CA, CDCA, and DCA are OATP1Bs substrates, as well as their respective glycine and taurine conjugates, and the conjugated bile acids (glycine and taurine) are superior to unconjugated bile acids as substrates for OATP1B1 and OATP1B3 [73]. In cynomolgus monkeys, rifampin treatment (18 mg/kg single oral dose) resulted in significantly elevated plasma levels of several bile acids, such as glycolic acid (GCA), glycochenodeoxycholic acid (GCDCA), and glycodeoxycholate (GDCA) compared with the vehicle control [62]. Similarly, plasma AUC of sulphate conjugates of BAs including GDCA-S, GCDCA-S, taurochenodeoxycholate (TCDCA-S), DCA-S, and taurodeoxycholate (TDCA-S) presented a robust dose-response to oral rifampicin (1, 3, 10, and 30 mg/kg) in cynomolgus monkeys [74]. The clinical use of bile acids as surrogate endogenous biomarkers for DDIs involving OATP1B1 and OATP1B3 has been explored in healthy volunteers. One study reported 20.3 fold increase of GCDCA-S after the single dose administration of rifampicin (600 mg, oral) [20], a finding that is in line with a study in which dose-dependent effects of rifampicin (150, 300, or 600 mg) on several putative biomarkers (bilirubin, CP-I, and bile acids) was examined [12]. These investigations suggest that a number of bile acids, including GCDCA-S, GCDCA-G, GDCA-G, GDCA-S, CDCA-24G, lithocholate sulfate (LCA-S), glycolithocholate sulfate (GLCA-S), and taurolithocholate sulfate (TLCA-S), can be further explored for the quantitative assessment of potential OATP1B-mediated DDIs in humans [13,17,18]. It is worth noting that bile acids are also substrates for other transporters (e.g., GCDCA-S is substrate of NTCP and OAT3), and often altered by disease conditions, which together attenuated the use of bile acids as a potential specific OATP1Bs function.

#### 2.1.4. Tetradecanedioate (TDA) and Hexadecanedioate (HDA)

The fatty acid dicarboxylates tetradecanedioate (TDA) and hexadecanedioate (HDA) have been identified as substrates of OATP1B1 as well as OAT1 and OAT3, and could potentially serve as endogenous biomarkers of OATP1B [7]. In rats, probenecid (a weak OATP1B inhibitor) increased plasma concentrations of HDA, and the tissue uptake clearance of deuterium-labelled HDA (*d*-HDA) in the liver was 16-fold higher than that in the kidney. The hepatic uptake clearance was reduced by 80% by probenecid, suggesting that HDA might be a biomarker for OATP1B that is minimally affected by urinary and biliary elimination in rats [75]. In healthy subjects, the administration of the OATP1B inhibitors rifampin (600 mg) or cyclosporin A (100 mg) also significantly increased plasma AUC of TDA and HDA [19,23,68], and the effects on HDA were dependent on the rifampicin dose (150, 300, or 600 mg) [12].

### 2.2. OAT1 and OAT3

The organic anion transporter OAT1 [*SLC22A6*] and OAT3 [*SLC22A8*] are mainly expressed on the basolateral membrane of renal proximal tubular cells and mediate cellular uptake of substrates from the blood into the kidney [1]. Inhibition of these transporters reduces drug renal clearance and can lead to DDIs. Taurine and GCDCA-S have been identified as endogenous OAT1/3 substrates using metabolomics analysis and DDI studies in healthy subjects receiving probenecid, a potent inhibitor of OATs. Along with in vitro evidence, taurine and GCDCA-S may potentially serve as endogenous biomarkers for assessing DDIs with these transporters [21]. However, the selectively of taurine and GCDCA-S as OAT1/3 endogenous biomarker is limited because of involvement of urine reabsorption with taurine and the interaction with other transporters (OATP1B1 and OATP1B3) for GCDCA-S. Recently, pyridoxic acid (PDA) and homovanillic acid (HVA) were identified as endogenous substrates of OAT1/3 using untargeted metabolomics analysis and DDI studies in cynomolgus monkeys receiving 40 mg/kg probenecid. In vitro study further confirmed PDA and HVA are substrates for human OAT1 and OAT3 [25]. Several most recent studies from healthy subjects suggested that PDA was a promising endogenous biomarker for OAT1/3 function sensitivity in response to weak, moderate, and strong OAT1/3 inhibitors [24,76]. In a recent study, Willemin et al. systemically tested the selectivity of PDA, HVA, GCDCA-S and taurine towards different renal transporters [22]. Using in vitro transporter overexpressing cell lines, this study demonstrated that PDA and HVA are substrates of OAT1/2/3, OAT4 [*SLC22A11*] (PDA only), MRP4 [*ABCC4*], GCDCA-S was a substrate of OAT3 and MRP2, and taurine was not a substrate of the tested transporters. In addition, the same study found that PDA was the most sensitive plasma biomarker in response to strong and selective OAT inhibitor probenecid (500 mg every 6 h), while GCDCA-S was the most sensitive OAT biomarker based on renal clearance [22]. Taken together, the fact that the inhibition of OATP1B1 and OATP1B3 by probenecid is unlikely to be remarkable [21], this study suggests that the combined monitoring of PDA and GCDCA-S from both urine and plasma should be recommended to tease out the involvement of OAT1/3 in DDIs [22]. Recently, metabolomics analyses of the serum of Oat1 and Oat3 knockout mice revealed remarkable changes in tryptophan derivatives involved in metabolism and signalling, suggesting that these metabolites can potentially be used as endogenous biomarkers to determine if drug candidates interact with OAT1 and/or OAT3 [77].

## 3. Organic Cation Transporters

The organic cation transporters (OCTs) are a group of poly-specific transporters with largely overlapping substrate selectivity, which include OCT1 [*SLC22A1*], OCT2 [*SLC22A2*], OCT3 [*SLC22A3*], OCTN1 [*SLC22A4*], OCTN2 [*SLC22A5*], MATE1 [*SLC47A1*], and MATE2-K [*SLC47A2*]. Their function is to transport organic cations, zwitterions, and some uncharged compounds as facilitated diffusion systems and/or antiporters, and they modulate the distribution of many endogenous compounds such as thiamine, creatinine, and neurotransmitters [78]. However, organic cation transporters also play an important role in the absorption, distribution, and excretion of hydrophilic drugs and therefore can be critically important contributors to DDIs [79]. Among the tested potential biomarkers of OCTs, *N*^1^-Methylnicotinamide (NMN) has been most extensively evaluated as endogenous biomarkers for the assessment of changes in the activity of renal OCT2/MATEs, while thiamine is widely discussed for OCT1 [29,31,32,80]. Given the overlapping substrate specificity of OCT2 and MATEs, many endogenous substrates proposed as biomarkers cannot be easily discriminated between these respective transporters (Table 1).

### 3.1. Thiamine

Thiamine, or vitamin B1, is an essential vitamin that is found at low levels in most foods [81]. Under physiologic conditions, elimination of thiamine is mainly extrarenal, although when given at high doses, renal excretion may become the principal path of elimination due to the saturation of other pathways [82,83]. The urinary elimination is predominantly associated with glomerular filtration, consistent with its low molecular weight and the lack of protein binding, although tubular secretion and reabsorption processes may also be probably involved. At physiologic conditions, the result is net reabsorption of thiamine [29,84], whereas under thiamine-deficient conditions, the renal excretion rate drops toward zero due to reabsorption [83]. When thiamine plasma concentrations are increased (>200 nM by i.v. administration) clearance increases to values close to estimated renal blood flow, which could be a sign of saturation of reabsorption while secretion processes remain unsaturated [83].

Thiamine is reported to be a substrate of MATE1 (*K*_m_ = 0.83–3.5 µM), MATE2K (*K*_m_ = 3.9 µM) [27,29,85], OCT1 (*K*_m_ = 0.78 µM), OCT2 (*K*_m_ = 0.75–59.9 µM) [27,86], thiamine transporter (THTR) 1 [*SLC19A2*] and THTR2 [*SLC19A3*] (*K*_m_ = 2.5 µM and 27 nM, respectively) [86,87] through in vitro transport studies. Its renal clearance is non-linear in humans and under normal conditions below the glomerular filtration rate (GFR), suggesting it undergoes active tubular reabsorption, possibly mediated by THTR1 and THTR2 [49]. Clinical evidence supporting thiamine as an endogenous biomarker to any of the above transporters is very limited, though multiple studies using transporter deficient mice suggested the role of transporters in thiamine disposition. An untargeted metabolomics study from healthy subjects revealed that renal clearance of thiamine was substantially reduced (70–84%) in the subjects treated with MATEs inhibitor pyrimethamine compared with the control group, which suggests that MATEs account for the efflux of thiamine [29]. Of note, thiamine was also evaluated in a preclinical transporter deficient mice model for the inhibitory potential of drugs toward OCT1 and OCT2, however, human data for this hypothesis are not yet available [80]. Compared to wild-type mice, levels of endogenous thiamine were 5.8-fold higher in Oct1/Oct2 double-knockout mice, while renal clearance of high-dose infusion of exogenous thiamine (intended to eliminate effects of renal reabsorption) was almost 79% lower [80]. β-oxidation and adenosine monophosphate-activated protein kinase activity were increased in the livers in Oct1^(–/–)^ mice, which could be indicative of OCT1 involvement in thiamine disposition [88]. In wild-type mice, the MATE inhibitor pyrimethamine reduced urinary excretion of endogenous thiamine by almost 70% [29] and renal clearance of exogenous thiamine by 58% [27,80]. The various transporters involved in the in vivo handling of thiamine suggests its clinical utility as an endogenous biomarker to mechanistically support for any particular DDI is very limited.

### 3.2. N^1^-Methylnicotinamide

*N*^1^-Methylnicotinamide (NMN) is produced as a result of tryptophan and niacin metabolism [89], and was previously identified as a substrate of OCT2 (*K*_m_ =300–318 µM), MATE1 (*K*_m_ = 301 µM), and MATE2K (*K*_m_ = 422 µM) in overexpressed HEK293 cells or Xenopus laevis oocytes [31,32,90,91]. The uptake of NMN in overexpressed HEK293 cells was inhibited by pyrimethamine with *K*i values of 9.4 µM (OCT2), 83 nM (MATE1), and 56 nM (MATE2K) and by trimethoprim with IC_50_ values of 134 µM (OCT2), 29 µM (MATE1), and 0.61 µM (MATE2K) as well as by other OCT2/MATE substrates and inhibitors such as metformin, tetraethyl ammonium (TEA), quinine, and cimetidine [32].

The contribution of transporters to the uptake of NMN is species-dependent [31], and plasma concentrations of NMN follow a circadian rhythm with higher plasma concentrations being observed in the morning and lower concentrations in the evening [92]. This could be a limitation in exploring the utility of NMN as a transporter biomarker, especially since studies involving the collection of single plasma samples commonly do not take into account the diurnal changes of NMN levels. Since NMN is not bound to plasma proteins in humans [93], it may be filtered freely in the glomerulus. In humans, NMN renal clearance is higher than the GFR, suggesting significant tubular secretion. Renal tubular reabsorption seems to also affect the clearance of NMN, in a saturable manner. Nonetheless, as a potential biomarker for renal OCT2/MATEs, renal clearance of NMN is of particular interest [84].

NMN has been explored as a potential endogenous OCT2/MATEs transporter biomarker in several human DDI studies [31,32]. In a study involving healthy subjects, renal clearance of NMN was significantly reduced in groups treated with pyrimethamine, in comparison to the control group, to the levels similar to GFR, suggesting that the active secretion of NMN was completely suppressed by pyrimethamine [49]. Other studies involving pyrimethamine and metformin in healthy subjects as a crossover study reported that NMN renal clearance was reduced in the presence of pyrimethamine in a way comparable to metformin renal clearance [31,32,36,94]. A positive correlation between the relative extent of the effect of trimethoprim on NMN renal clearance correlated positively, and the effect of trimethoprim on metformin renal clearance was also reported, which adds to the credibility that NMN serves as a biomarker of renal MATE function [31,32]. Although renal clearance of NMN was reduced, its plasma concentrations were not increased by pyrimethamine or trimethoprim. This suggests that pyrimethamine and trimethoprim may affect NMN formation or metabolism through different pathways. It is recommended to control known confounders such as study design, relevant concomitant medication, and standardized food and water intake, as well as physical activity, when using NMN for the assessment of a new molecular entity [84,95].

### 3.3. N^1^-Methyladenosine

Metabolomics data using wild-type and Oct1/2 double knockout mice identified *N*^1^- Methyladenosine (m^1^A) as a novel OCT2 substrate [6]. In vitro transport studies confirmed that m^1^A is a substrate of mouse Oct1, Oct2, Mate1, human OCT1, OCT2, and MATE2-K, but not human MATE1 [6]. Urinary excretion is responsible for 77% of the systemic elimination of m^1^A in mice and renal clearance of exogenously administered m^1^A is decreased in Oct1/2 double knockout or by Mate1 inhibition by pyrimethamine to near the glomerular filtration rates while plasma concentrations are increased [6]. In cynomolgus monkeys, a single dose of DX-619, an OCT2/MATE2K inhibitor increased the AUC of m^1^A (1.72-fold) as well as metformin (2.18-fold) [6]. In humans, renal clearance of m^1^A in healthy individuals is higher than the GFR, which supports the significant contribution of tubular secretion to urinary excretion. With low diurnal and inter-individual variation in plasma concentrations in healthy volunteers, m^1^A could be further explored as a surrogate probe for the evaluation of DDIs involving OCT2/MATE2K [5].

### 3.4. Carnitine

Carnitine, and its derivatives acetylcarnitine and propionyl carnitine showed a substantial and consistent decrease of urinary excretion in both mice and healthy volunteers in untargeted metabolomics analysis when pyrimethamine was given as a MATE inhibitor. In healthy volunteers treated with metformin at a micro dose or therapeutic dose, renal clearance of carnitine and acetylcarnitine was decreased by 90–94% and 87–91%, respectively, when pyrimethamine was co-administered [29], and pyrimethamine also reduced carnitine renal clearance by 62% in mice [29]. However, since in vitro studies with HEK293 cells overexpressing MATE1 or MATE2K did not show an increase in carnitine uptake compared with control [85,91], the molecular mechanisms responsible for the effect of pyrimethamine on clearance of carnitine and its metabolites remains unclear.

The urinary excretion of both carnitine and acetylcarnitine is reduced in Oct1/Oct2 double-knockout mice in comparison with wild-type mice, and carnitine was found to be a substrate of OCT2, which suggests the involvement of OCT2 in the renal secretion of carnitine and possibly acetylcarnitine [84]. Nonetheless, in vivo data on this role in humans is lacking [4]. For acylcarnitine, an association with OCTN1, UGT1A1, and carnitine palmitoyltransferase 1 genes have been reported and acylcarnitine is also an in vitro substrate of OCTN2, taken together, these results limit the selectivity of acylcarnitine as a biomarker for OCT2 [96].

Isobutyryl-carnitine (IBC) has also been recently suggested as a potential OCT1 biomarker from this family, and while genomic data in mouse and humans with active OCT1 genotypes suggests that is a substrate of OCT1 with carriers of high-activity OCT1 genotypes having almost 3-fold higher IBC concentrations in blood and 2-fold higher in urine compared to deficient OCT1, more specific data for IBC are lacking [97,98].

### 3.5. Creatinine

Creatinine is produced during muscle metabolism and excreted primarily through glomerular filtration in the kidney. In vitro, creatinine has low affinity as a substrate of OCT2, MATE1, MATE2K, and OCT3 [99,100,101], and about 10−40% of creatinine is excreted through active tubular secretion in kidneys involving OCT2, OAT2, MATE1, and MATE2K. Based on in vitro and in vivo studies on correlation analyses of the inhibition of OCT2, MATE1, and MATE2K and clinically observed changes in serum creatinine (SCr) or creatinine clearance, several compounds were positively predicted to cause OCT2/MATE-mediated DDIs. However, the changes in serum creatinine associated with OCT2/MATE interactions are usually not significant enough to justify its use as a biomarker. For example, in the case of cimetidine co-administered with metformin, and ranitidine co-administered with procainamide or triamterene, interactions were observed with no apparent change in serum creatinine levels. Furthermore, numerous confounding factors such as weight, health condition, gender, age, muscle metabolism, and diet affect creatinine clearance, which collectively limit its use as a biomarker for OCT2/MATE-mediated DDIs [49].

### 3.6. Others

Dopamine is a MATE substrate. As an in vitro MATE inhibitor, its renal excretion is reduced in Mate1 knockout mice or with the administration of imatinib [40,102]. Dopamine is also a substrate of OCT2 in vitro [39], but since it is synthesized in renal proximal tubule cells, the relationship between OCT2 activity and urinary dopamine excretion might be limited [40]. Human data on dopamine use as a MATE biomarker is lacking.

The amino acid, tryptophan, is an OCT1 and OCT2 substrate, which was identified in an untargeted metabolomics analysis of urinary samples of 21 subjects who had participated in the clinical trial on metformin pharmacokinetics, in subjects carrying different functional variants in the OCT2 gene, *SLC22A2* [103]. A positive linear association was previously observed between urinary tryptophan and pharmacokinetic parameters of metformin, such as renal clearance and renal secretory clearance. In vivo data in humans and more DDI data on inhibitors are needed to justify the use of tryptophan as a potentially useful biomarker [49].

## 4. Conclusions and Future Directions

The family of solute carriers is increasingly recognized as having an important role in the pharmacokinetics and pharmacodynamics profiles of many prescription drugs, and as a site of relevance to DDIs. Mechanistically, most of these DDIs can be linked directly to effects of a select set of xenobiotic transporters that are critically important in mediating drug disposition patterns. These transporters include OATPs, OATs, OCTs, and MATEs, and current regulatory requirements [104] propose an integrated approach to assess DDIs and the potential of a new molecular entity, however, the current in vitro approach is rather conservative and may result in false-positive prediction and subsequently large number of traditional clinical DDIs studies using probe drugs. There is a need to an efficient and cost-effective solution and identify alternative approaches that could help facilitate the prediction of DDIs in early stages of drug development. Measurement of endogenous biomarkers could be a practical and affordable approach as a substitute for traditional DDI studies using probe drugs, especially when clinically efficacious doses are not yet known.

Such potential biomarkers should be thoroughly investigated and validated and should include considerations related to selectivity and specificity (determining exactly which transporters are involved in a biomarker’s distribution), sensitivity (the magnitude of effect should be significant enough to allow differentiation between weak, moderate, and strong inhibitors), reliability, and reproducibility (the observed change should be predictive of clinically relevant DDIs, and not due to inter and intra-individual variabilities due to sex, age, disease state, circadian rhythm, diet or exercise) [49,105]. Although many potential biomarker candidates for transporters have been identified in recent years using various approaches and some have even been tested in clinical studies, none have yet conclusively demonstrated utility as a substitute for clinical DDIs studies. One reason for this is that most potential biomarkers have not been tested in connection with weak transporter inhibitors, where anticipated biomarker changes are lower compared with more sensitive exogenous probe substrates. These considerations demand special attention to the time course of response of a perpetrator drug on a biomarker to generate reliable data. The use of a standard cut-off value to determine the change in biomarker exposure may not be possible to all the tested transporters due to variability in the magnitude of interaction, instead, a dose dependent increase of exposure of the biomarker in response to a test inhibitor is recommended as a positive implementation of an interaction with the transporter of interest. Another reason might be the relatively low selectivity/specificity of presently identified endogenous biomarkers. A better mechanistic understanding of the formation, disposition, and elimination of the biomarker, such as contribution of genetic polymorphisms to the biomarker disposition, would greatly facilitate interpretation of the biomarker DDIs data.

One particularly fruitful area of research that could help resolve the above problems is related to the implementation of a bottom-to-top approach that involves the selection of endogenous transporter substrates from untargeted metabolomics approaches in transporter-deficient and humanized transgenic mice in the presence and absence of specific inhibitors. This would allow for the identification of putative biomarkers in a more unbiased manner that does not depend on top-to-bottom strategies that are initiated by observations in human subjects where the likelihood of identifying selective and specific biomarkers is intrinsically compromised. In such bottom-up approaches, substrates with the most robust differences can be more easily identified and subsequently used to ensure that a sufficient degree of sensitivity is achieved. Another useful tool that requires further investigation is the application of endogenous biomarker cocktails to highlight the individual and collective importance of multiple transporters simultaneously in the context of DDIs prediction [49]. Careful consideration of clinical trial design is also required in order to ensure sufficient reliability of the generated data and, generally, studies should be designed in a manner that minimizes confounding factors and increases statistical power, for example by employing randomized crossover designs. Unfortunately, many biomarker-driven clinical DDIs studies published to date suffer from small sample sizes in relation to anticipated or observed effect sizes, as well as from a host of other potentially confounding factors that may have influenced their outcome. Most important among these are (unknown) environmental variables and physiologic factors that may affect expression of the transporters of interest, the use of healthy volunteers that may not adequately represent the target population of patients, and confounding links to other transporter genes or variants of putative relevance to drug disposition pathways.

Although the use of cocktail strategies together with multiple biomarkers opposed to testing single biomarkers to predict certain DDIs has been proposed and may have clinical importance, this remains to be clarified for most perpetrator drugs. In addition, more detailed investigations into the influence of ethnicity and racial ancestry on the functional utility of particular transporter biomarkers, and on the basal function and expression of relevant transporters, is urgently required. Despite these reservations, it is anticipated that, over the next decade, the importance of transporter biomarkers and their role in DDI prediction will be more clearly defined, and that continued investigation in this area will likely have a profound impact on attempts to refine treatment regimens involving polypharmacy with transporter substrates.

## Figures and Tables

**Table 1:** List of endogenous biomarkers (candidates) of drug transporters for DDI evaluation.

Compounds	Pathway	Transporters	In Vitro Results		Clinical DDI with Known Inhibitors		References
			Expression System	Transport Kinetics	Inhibitors/Genotype	PK Change	
Bilirubin Direct bilirubin (D)/total bilirubin (T)	Haemoglobin and Porphyrin Metabolism	OATP1B1 OATP1B3	HEK293	*K*_m_ = 0.16 μΜ	Rifampicin 300 mg, sd, p.o.	AUCR: 2.3 (D), 1.6 (T)	[10,11,12,13]
			*X. laevis oocytes*	*K*_m_ = 0.0391 μΜ			
					Rifampicin 600 mg, sd, p.o.	AUCR: 3.5 (D), 1.7 (T)	
					Rifampicin 150, 300, 600 mg, sd, p.o.	AUCR_0–24h_ (D): 1.26, 1.99, 2.77	
Coproporphyrin I (CPI)/Corproporphyrin III (CPIII)	Haemoglobin and Porphyrin Metabolism	OATP1B1	CHO	*K*_m_ = 0.13 μΜ (CPI) and 0.22 μΜ (CPIII)	Rifampicin 300 mg, sd, p.o.	CPI AUCR: 3.0	[12,13,14,15,16,17,18,19]
					Rifampicin 600 mg, sd, p.o.	CPI AUCR: 4.0 CPIII AUCR: 3.3	
					Rifampicin 150, 300, 600 mg, sd, p.o.	CPI AUCR_0–24h_: 1.54, 2.33, 3.67	
		OATP1B3	HEK293	*K*_m_ = 3.95 μΜ (CPI) and 1.55 μΜ (CPIII)	Cyclosporine A 100 mg, dd, p.o.	CPI AUCR: 1.7 CPIII AUCR: 1.9	
					Paclitaxel 200 mg/m^2^, sd, i.v.	CPI AUCR: 2.8 CPIII AUCR: 3.1	
		OATP2B1	CHO	*K*_m_ = 0.31 μΜ (CPIII)	Glecaprevir/pibrentasvir 300/120 mg fixed dose	CPI AUCR_0–16h_: 1.39 CPIII AUCR_0–16h_: 1.01	
GCDCA-S CDCA-24G Other Bile Acids	Bile Acid Metabolism	OATP1B1	HEK293	GCDCA-S: *K*_m_ = 9.95 μΜ CDCA-24G: *K*_m_ = 11.5 μΜ	OATP1B1/OATP1B3 Rifampicin 600 mg, sd, p.o.	AUCR: 20.3 (GCDCA-S)	[12,13,17,20,21,22]
					Rifampicin 300 and 600 mg, sd, p.o.	AUCR: 4.3 and 10 (GCDCA-S), 1.5 and 1.7 (CDCA-24G)	
		OATP1B3	HEK293	GCDCA-S: *K*_m_ = 5.23 μΜ CDCA-24G: *K*_m_ = 16.5 μΜ			
					Rifampicin 150, 300, 600 mg, sd, p.o.	AUCR_0–24h_: 2.28, 5.87, 15.9 (GCDCA-S); 2.18, 5.41, 14.6 (GCDCA-G); 1.49, 2.00, 3.43 (CDCA-24G)	
					Paclitaxel 200 mg/m^2^, sd, i.v.	AUCR: GCDCA-S: 2.9 GDCA-S: 4.0 LCA-S: 2.0 GLCA-S: 2.2 TLCA-S: 2.0 GCDCA-G: 2.5 GDCA-G: 2.5 CDCA-24G: 3.0	
		OAT3	HEK293	GCDCA-S: *K*_m_ = 64.3 μΜ	OAT1/OAT3 Probenecid 500, 750, and1500 mg, sd, p.o.	AUCR: 1.06, 1.00, and 1.37 (GCDCA-S)	
					Probenecid 500 mg qid, p.o.	AUCR_0–24h_: 1.9 (GCDCA-S)	
Tetradecanedioate (TDA)/Hexadecanedioate (HDA)	Fatty Acid Metabolism	OATP1B1	HEK293	IC_50_ = 4.0 μΜ (TDA) and 1.6 μΜ (HDA)	Rifampicin 600 mg, sd, p.o.	AUCR (TDA): 3.2 AUCR (HDA): 2.2	[7,12,19,23]
					Cyclosporine A 100 mg, dd, p.o.	AUCR (TDA): 1.8 AUCR (HDA): 1.9	
		OAT1/OAT3	HEK293	Positive	Rifampicin 150, 300, 600 mg, sd, p.o.	AUCR (TDA): 1.36, 2.01, 2.30 AUCR (HDA): 1.58, 2.30, 2.98	
Taurine	Taurine Metabolism	OAT1	HEK293	*K*_m_ = 379 μΜ	Probenecid 500, 750, and1500 mg, sd, p.o.	AUCR: 0.97, 0.98, 1.02	[21,22]
		OAT3	HEK293	Negative			
					Probenecid 500 mg qid, p.o.	AUCR_0–24h_: 1.1	
Pyridoxic acid (PDA)	Vitamin B6 Metabolism	OAT1/OAT3	HEK293	Positive	Probenecid 1000 mg, sd, p.o.	AUCR: 3.3	[22,24,25]
					Probenecid 500 mg qid, p.o.	AUCR_0–24h_: 3.7	
Homovanillic acid (HVA)	Tyrosine metabolism	OAT1/OAT3	HEK293	Positive	Probenecid 1000 mg, sd, p.o.	AUCR: 2.0	[22,24,25]
					Probenecid 500 mg qid, p.o.	AUCR_0–24h_: 2.1	
Thiamine	Thiamine Metabolism	OCT1	HEK293	*K*_m_ = 780 μΜ	OCT2/MATEs Pyrimethamine 50 mg, sd, p.o.	AUCR: 1.0	[26,27,28,29]
		OCT2	HEK293	*K*_m_ = 59.9–750 μΜ			
		OCT3	HEK293	*K*_m_ = 443 μΜ			
		MATE1	HEK293	*K*_m_ = 3.9–44.7 μΜ			
		MATE2-K	HEK293	*K*_m_ = 3.9–5.2 μΜ			
*N*^1^-Methylnicotinamide (NMN)	Nicotinate and Nicotinamide Metabolism	OCT2	HEK293	*K*_m_ = 318 μΜ	OCT2/MATEs Pyrimethamine 50 mg, sd, p.o.	AUCR: 0.84	[30,31,32]
		MATE1	HEK293	*K*_m_ = 301 μΜ			
					Trimethoprim 200 mg, bid, p.o.	AUCR: 1.00	
		MATE2-K	HEK293	*K*_m_ = 422 μΜ			
*N*^1^-Methyladenosine (m^1^A)		OCT1/2, MATE2-K	HEK293	Positive	DX-619	AUCR: 1.72	[6]
Carnitine/ Acetylcarnitine	Amino acid derivative	OCT2, OCTN1, OCTN2			Pyrimethamine 50 mg, sd, p.o.	Reduced renal clearance by 90%	[29]
Creatinine	Creatine Metabolism	OCT2	S2, HEK293, MDCKII	*K*_m_ = 1.86–18.8 mΜ	OCT2/MATEs Cimetidine 400 mg, bid, p.o.	AUCR: 1.1	[33,34,35,36,37,38]
		MATE1	HEK293, MDCKII	*K*_m_ = 10.2 mΜ			
		MATE2-K	HEK293, MDCKI	*K*_m_ = 21.6 mΜ	Pyrimethamine 50–100 mg, sd, p.o.	AUCR: 1.2–1.3	
		OAT2	HEK293, MDCKII	*K*_m_ = 795–986 μΜ			
					Dolutegravir 50 mg, qd/bid, p.o.	AUCR: 1.1	
					DX-619 800 mg, qd, p.o.	AUCR: 1.3	
		OCT3	MDCKII	*K*_m_ = 1.32 mΜ	Trimethoprim 200 mg, bid, p.o.	AUCR: 1.2	
		OAT3/OAT4	S2	Positive			
Dopamine	Tyrosine Metabolism	MATE1	HEK293	*K*_m_ = 0.56 mM			[39,40]
		MATE2-K	HEK293	*K*_m_ = 2.48 mΜ			
		MATE1	HEK293	*K*_m_ = 0.53 mM			
		OCT2	*X. laevis oocytes*	*K*_m_ = 0.39 mM			
Tryptophan	Tryptophan Metabolism	OCT2	*X. laevis oocytes*	IC_50_ = 6.11 mM	*SLC22A1* c.808G > T(rs316019) homo	CLr Ratio: 0.64	[41]

AUCR, ratio of area under the plasma concentration time curve; CLr, renal clearance; sd, single dose; dd, double dose; qd, quaque die; bid, bis in die; qid, quater in die; Positive, significant activities compared with control; Negative, no significant activities compared with control.

## Data Availability

Not available.
